# Comparative genomics of chondrichthyan Hoxa clusters

**DOI:** 10.1186/1471-2148-9-218

**Published:** 2009-09-02

**Authors:** John F Mulley, Ying-Fu Zhong, Peter WH Holland

**Affiliations:** 1 Department of Zoology, University of Oxford, South Parks Road, Oxford, OX1 3PS, UK; 2 Center for Bioinformatics, National Laboratory of Protein Engineering and Plant Genetic Engineering, College of Life Sciences, Peking University, Beijing 100871, PR China

## Abstract

**Background:**

The chondrichthyan or cartilaginous fish (chimeras, sharks, skates and rays) occupy an important phylogenetic position as the sister group to all other jawed vertebrates and as an early lineage to diverge from the vertebrate lineage following two whole genome duplication events in vertebrate evolution. There have been few comparative genomic analyses incorporating data from chondrichthyan fish and none comparing genomic information from within the group. We have sequenced the complete Hoxa cluster of the Little Skate (*Leucoraja erinacea*) and compared to the published Hoxa cluster of the Horn Shark (*Heterodontus francisci*) and to available data from the Elephant Shark (*Callorhinchus milii*) genome project.

**Results:**

A BAC clone containing the full Little Skate Hoxa cluster was fully sequenced and assembled. Analyses of coding sequences and conserved non-coding elements reveal a strikingly high level of conservation across the cartilaginous fish, with twenty ultraconserved elements (100%,100 bp) found between Skate and Horn Shark, compared to three between human and marsupials. We have also identified novel potential non-coding RNAs in the Skate BAC clone, some of which are conserved to other species.

**Conclusion:**

We find that the Little Skate Hoxa cluster is remarkably similar to the previously published Horn Shark Hoxa cluster with respect to sequence identity, gene size and intergenic distance despite over 180 million years of separation between the two lineages. We suggest that the genomes of cartilaginous fish are more highly conserved than those of tetrapods or teleost fish and so are more likely to have retained ancestral non-coding elements. While useful for isolating homologous DNA, this complicates bioinformatic approaches to identify chondrichthyan-specific non-coding DNA elements

## Background

**Figure 1:**
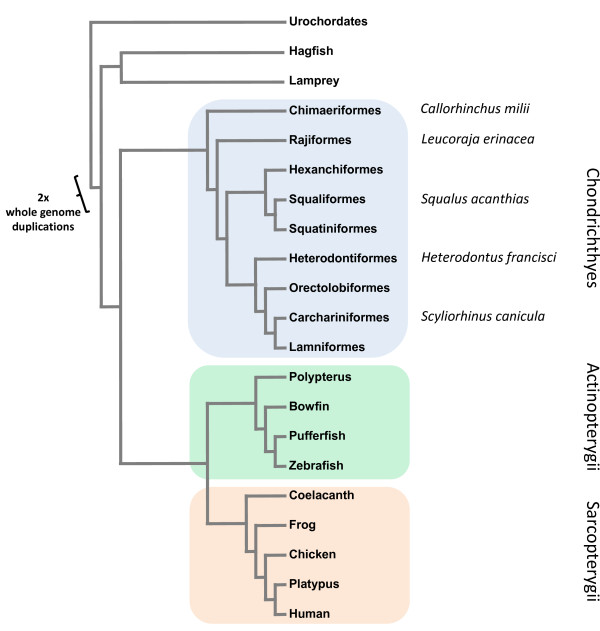
**Vertebrate relationships**. A cladogram showing vertebrate evolutionary relationships. Phylogeny of the cartilaginous fish is based on Naylor *et al*. [14].

The Chondrichthyes (cartilaginous fish) are one of the three extant major clades of jawed vertebrates and comprise two sister-groups; the elasmobranchs (sharks, skates and rays) and the holocephalans (chimeras and ratfish - Figure 1). Although relatively species-poor by comparison to their osteichthyan (bony vertebrate) relatives (~970 species of chondrichthyan, ~55,600 species of bony vertebrates [1]), they occupy an important phylogenetic position as the sister-group to all other jawed vertebrates and as one of the first lineages to diverge after the two rounds of whole genome duplications that occurred early in vertebrate evolution [2]. Despite this, there have been relatively few studies dealing with chondricthyan genomics. Those that have been carried out have usually used cartilaginous fish as the outgroup to studies of bony vertebrates [3,4]. One problem is the large size of many chondricthyan genomes, which range from 1.5 to 16.7 Gb with a median value of 7.1 Gb or roughly twice the size of the human genome [5]. Venkatesh *et al*. [6] completed a 1.4× coverage draft survey of the Elephant Shark (*Callorhinchus milii*) genome (a member of the holocephalans, the sister group of the true sharks and rays), having chosen this species because of its smaller genome. Additionally, a Little Skate (*Leucoraja erinacea*) genome project has been approved by the National Human Genome Research Institute. However, there is currently little information available on patterns of genome evolution within the cartilaginous fish. To start to rectify this omission, we undertook the sequencing and analysis of a complete Hox cluster from the Little Skate.

The Hox genes play important roles in determination of anterior-posterior patterning during embryonic development [7]. Their clustered organisation in the genome is a consequence of their origin from tandem gene duplications and is intimately associated with their expression during development. To date, all invertebrate lineages have been found to have only a single set of Hox genes, generally arranged in a single Hox cluster. In contrast, vertebrates have multiple more compact clusters, with four being present in tetrapods (*Hoxa*, *Hoxb*, *Hoxc *and *Hoxd *[8]) as a result of two rounds of whole genome duplication at the base of the vertebrates. Teleost fish have up to eight clusters and, within teleosts, the salmonids have at least thirteen as the result of additional genome duplications [9-11]. Furthermore, teleost Hox clusters are known to evolve at a faster rate than those of tetrapods, making it difficult to identify ancient conserved non-coding sequences [12]. Two Hox gene clusters (*Hoxa *and *Hoxd*) have been fully sequenced from a chondrichthyan (the Horn Shark *Heterodontus francisci *[3,4]). Because of this, and the availability of the complete sequence of the Hoxa cluster of the Senegal bichir (*Polypterus senegalus *[13]) - a member of the earliest diverging lineage of ray-finned fish and therefore unaffected by the whole genome duplications which increased Hox cluster number in teleosts - we chose to analyse the Hoxa cluster of Little Skate (*Leucoraja erinacea*), a member of the oldest extant lineage of Elasmobranchs [14]. Together with the data from the more recently diverging Horn Shark and outgroup Holocephalan sequences we can for the first time carry out a comparative genomic analysis within the cartilaginous fish.

## Results and Discussion

### Size of Chondrichthyan Hoxa clusters

**Figure 2:**
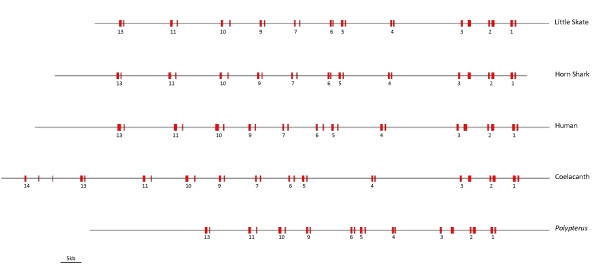
**Organisation of Hoxa clusters in vertebrates**. This figure includes the two complete chondricthyan sequences (Little Skate and Horn Shark), two members of the sarcopterygian clade (Human and Indonesian coelacanth, *Latimeria menadoensis*) and an actinopterygian (ray-finned fish), *Polypterus senegalus*. The Skate and Horn Shark clusters are remarkably similar in overall length and intergenic distance.

**Figure 3:**
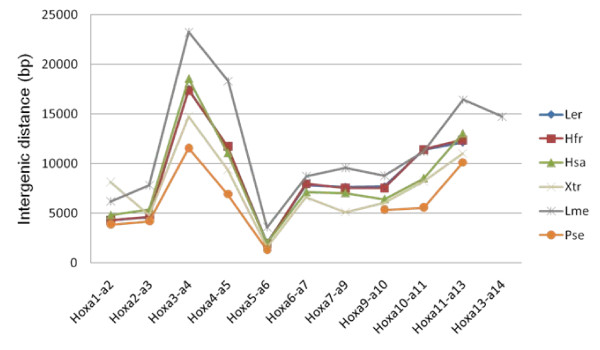
**Intergenic distance in the Hoxa clusters of several vertebrates**. The Little Skate and Horn Shark intergenic distances are virtually identical. Ler -- Little Skate, Hfr -- Horn Shark, Hsa -- human, Xtr, *Xenopus tropicalis*, Lme -- Indonesian coelacanth, Pse -- *Polypterus senegalus*.

The 133, 863 bp sequence of the Little Skate BAC clone 0081H20 (submitted to Genbank under accession # FJ944024) contains the full Hoxa cluster as deduced by comparison to the published Horn Shark and Elephant Shark clusters [3,4,6], as well as those of other vertebrates (Figure 2). Although coelacanth has a *Hoxa14 *gene, we do not believe that this gene is present in either Skate or Horn Shark because of the distribution of conserved non-coding elements between the three species. Thus a conserved element found 5' to *Hoxa14 *in coelacanth is present 5' to *Hoxa13 *in Little Skate and Horn Shark. The distance from the start codon of *Hoxa13 *to the stop codon of *Hoxa1 *comprises 104,602 bp, strikingly similar to the 105,289 bp figure for the Horn Shark despite the genome size of the Horn Shark being around twice the size of that of Little Skate (6.65-8.6 Gb for *Heterodontus francisci*; 3.4 Gb for *Leucoraja erinacea *[5]). This similarity is also reflected in the intergenic distances within the clusters themselves (Figure 3). If the larger size of the Horn Shark genome is due to an expansion of repetitive DNA, then this is clearly not reflected in the overall size of the Hoxa cluster. Indeed gnathostome Hox clusters are renowned for being free of repetitive DNA [15] (although see [16] for an exception). It is currently not possible to determine the total length of the Hoxa cluster in the elephant shark, since the draft genome sequence has relatively short contigs [6]. However, several contigs contain the full coding sequence of single Hox genes so for these genes it is possible to compare gene length between the three chondrichthyans (Table 1). In these cases, the Horn Shark and Little Skate are virtually identical, whilst the Elephant Shark typically has slightly larger total gene lengths.

**Table 1:** Gene structure of the three Hoxa genes for which there are complete data in the Elephant Shark genome

Gene	Gene length (bp)											
	*L. erinacea*				*H. francisci*				*C. milli*			
	Exon 1	Intron	Exon 2	Total	Exon 1	Intron	Exon 2	Total	Exon 1	Intron	Exon 2	Total
Hoxa4	412	347	326	1085	412	349	326	1087	403	456	308	1167
Hoxa5	577	469	251	1297	577	473	251	1301	595	460	251	1306
Hoxa6	430	202	260	892	430	202	260	892	430	224	260	914

The Little Skate and Horn Shark values are remarkably similar, whilst the Elephant Shark typically has larger values, despite its smaller overall genome size.

### Sequence conservation in Chondrichthyan Hoxa clusters

**Figure 4:**
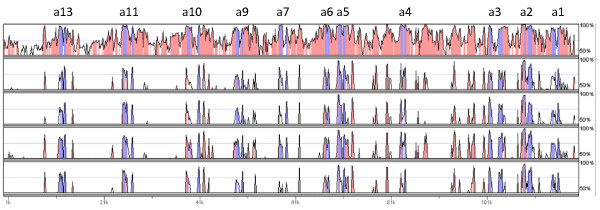
**Sequence conservation in Hoxa clusters**. VISTA plot [31] comparing the Little Skate Hoxa cluster (base sequence) with the orthologous regions in other vertebrates (from top to bottom: *Heterodontus francisci*, *Homo sapiens*, *Xenopus tropicalis*, *Latimeria menadoensis *and *Polypterus senegalus*). Shading indicates regions of 100 bp or more that are ≥ 70% identical in either exons (blue) or intergenic/intronic regions (pink).

Within the Little Skate Hoxa cluster, only 9522 bp codes for protein (compared to 9525 bp in Horn Shark). Hox gene coding sequence is typically around 95-98% identical at the amino acid level between the two species. Turning to non-coding DNA, an alignment of the Little Skate Hoxa cluster with the orthologous regions of other vertebrates (Figure 4) reveals a striking level of sequence identity between the two elasmobranchs even at reasonably stringent (but biologically arbitrary) alignment conditions of ≥ 70% identity over a 100 bp window. This is especially surprising given that the skates are thought to be the sister-group to all other extant elasmobranchs [14] and therefore must have diverged from them in the late Triassic or early Jurassic at least 200 million years ago. Even if skates and rays are not the sister group to other elasmobranchs, the divergence time of Little Skate and *Heterodontus *must still be at least 180 million years ago, based on the age of the oldest fossil Heterodontids and Batoids (skates and rays) from the Toarcian Stage of the Early Jurassic [17]. A comparable figure would be the divergence of the marsupial and human lineages around 162-191 million years ago [18].

### Ultraconserved regions in chondrichthyan Hoxa clusters

**Figure 5:**
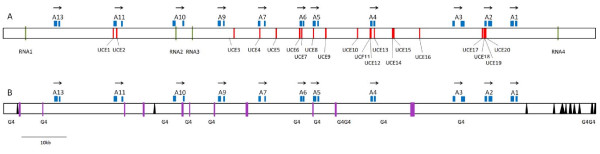
**(a). Organisation of UCEs and repetitive DNA in the Little Skate Hoxa cluster**. (a) Location of ultraconserved non-coding elements (red) conserved at 100% over 100 bp or more between Little Skate and Horn Shark in relation to the Hox genes (blue) and (b) location of repetitive elements in the Little Skate Hoxa cluster. Simple sequence repeats are represented as purple bars, LINEs and SINEs are shown with black triangles and potential G-quadruplex forming regions are indicated by 'G4'. The positions of the four predicted non-coding RNAs are also indicated (green).

Ultraconserved elements (UCEs) were originally defined as regions of 200 bp or more conserved at 100% identity between human and rodent genomes [19]. Recently, Stephen *et al*. [20] identified a large number of ultraconserved elements that are 100% identical over ≥ 100 bp in at least three placental mammals and Wang *et al*. [21] found that 41% of these UCEs were present in the Elephant Shark genome at an average identity of 79.8%. Of the 13,736 UCEs identified by Stephen *et al*. [20], thirty are located in the human Hoxa locus (UCEs El11507- El11536, I. Makunin, personal communication) and of these, fifteen are also found in Little Skate (at least 75% conserved with an uninterrupted stretch of at least 20 bp at 100%). With the complete sequence known for the Hoxa cluster of two species of chondricthyan, it is now also possible to look for UCEs conserved within the cartilaginous fish. We find 20 regions of ≥ 100 bp that are conserved at 100% identity between Little Skate and Horn Shark, nine of which overlap with coding regions and two of which are conserved at 100% identity over 200 bp (Figure 5a), of these, 14 are identifiable (but not 100% conserved) in the Hoxa clusters of a sarcopterygian (human) and a non-duplicated actinopterygian (*Polypterus*). This very high degree of conservation of ultraconserved elements between Little Skate and Horn Shark is surprising given the length of time that has passed since the divergence of these two elasmobranchs. By comparison, there are only three UCEs conserved absolutely between the Hoxa clusters of human and marsupials (both Platypus and Opossum), species that have been separated for approximately the same period of time. If the Elephant Shark genome is taken to be approximately 75% complete [21] one would expect to find ~15 of the 20 UCEs conserved between Skate and Horn Shark in this species. In fact, only three UCEs (3, 12 and 16) are missing from the current genome assembly, with the remaining 17 generally being present at 95-98% identity and five (UCEs 4, 10 and 13-15) present at 100% identity. Again, this level of conservation is surprising since holocephalans have been separated from the elasmobranchs for at least 375 million years (based on the oldest Holocephalan fossils from the Frasnian stage of the late Devonian [17]). A comparable figure for the human genome would be the divergence of human and frog (*Xenopus tropicalis*); species which have been separated for at least 330-350 million years and in which only a single Hoxa ultraconserved element at 100% over ≥ 100 bp is found. If the conservation threshold is lowered to 90%, then there are still only nine elements conserved between human and frog, five of which overlap with Hox coding sequences.

### Predicted non-coding RNA in chondrichthyan Hoxa clusters

**Figure 6:**
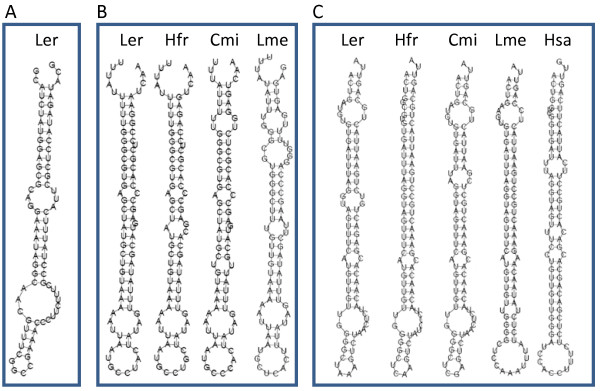
**Predicted non-coding RNAs (ncRNA) derived from the Little Skate Hoxa cluster**. Non-coding RNA1 (A) is found only in *Leucoraja erinacea*. Non-coding RNA2 (B) is located in the intron of the Hoxa10 gene and is found only in Little Skate, Horn Shark, Elephant Shark and Indonesian coelacanth. Non-coding RNA3 (C) is mir196b and is found in all vertebrates examined to date. Ler -- Little Skate, Hfr -- Horn Shark, Cmi - Elephant shark, Hsa -- human, Lme -- Indonesian coelacanth.

Vertebrate Hox clusters are known to contain several microRNAs (mir10a and mir10b in the Hoxb and Hoxd clusters; mir196a1, mir196a2 and mir196b in the Hoxa, Hoxb and Hoxc clusters) which are known to be involved in anti-sense regulation of Hox genes both within and across clusters [22]. The microRNA located in the Hoxa cluster (mir196b) is known to regulate *Hoxa7 *in the mouse through a reduction of protein level, rather than mRNA destabilisation [23] and has several potential binding sites in the 3'UTR of the *Hoxa7 *gene. There are four potential mir196b binding sites in the 3' UTR of Horn Shark *Hoxa7 *[24] and these are also found in the Little Skate sequence, although there is also an additional site at the beginning of the Skate *Hoxa7 *3' UTR. Bioinformatic identification of microRNA targets is complicated by the fact that the miRNA-target binding can include mismatches and gaps and often involves only a small part of the mature miRNA [25]. Interestingly, the *Polypterus senegalus *HoxA cluster has retained mir196b despite the loss of the *Hoxa7 *gene, lending support to the suggestion that mir196b has other targets within vertebrate Hox clusters [22]. We have identified four putative non-coding RNAs (ncRNAs - Figure 6) in the Little Skate BAC sequence through the prediction of stable stem-loop secondary structures. One predicted RNA is the known mir196b, indicating that the analysis can reliably recover ncRNAs. The second predicted RNA is located upstream of *Hoxa13 *and is found only in the Little Skate. The third is located within the intron of *Hoxa10 *and is conserved between Little Skate, Horn Shark, Elephant Shark and Indonesian Coelacanth, but not in teleosts or tetrapods. The final predicted ncRNA is more problematic, having an unusual secondary structure in skate and an even more unusual structure in Elephant Shark (not shown). Closer examination of this RNA suggests that it may not be functional, since it is located near to several repetitive elements (Figure 5b) in both Skate and Elephant Shark and the first 30 bp (which are also the last 30 bp due to the stem-loop structure) show numerous strong hits to many cartilaginous fish sequences on GenBank (including sequences from *Callorhinchus*, *Triakis*, *Heterodontus*, *Squalus*, *Ginglymostoma *and *Bathyraja*) as well as a very large number of hits to the Elephant Shark genome sequence. It is possible that this prediction actually reflects two repetitive elements in opposite orientations suggestive of a stable stem-loop structure.

### Repetitive DNA

Vertebrate Hox clusters are usually free of complex repeats. However, the Little Skate Hoxa cluster contains a 109 bp region between *Hoxa11 *and *Hoxa10 *which shows high (84%) identity to the Deu-domain and 3'-tail region of SacSINE1 identified by Nishihara *et al*. [26] in the dogfish shark (*Squalus acanthias*). SacSINE1 is a 463 bp tRNA-derived SINE related to the SINE3 family of zebrafish and thought to be derived from the L2 clade of LINEs. The Little Skate SINE fragment lacks the 5' promoter for transcription by RNA III polymerase, indicating that it is a non-functional retroposon although it may have accumulated some other function which accounts for the degree of sequence conservation to SacSINE1. Alternatively, the Little Skate SINE may represent a recent retroposition into the Hox cluster which has been inactivated by the loss of the 5' promoter region. The orthologous region of the Horn Shark Hoxa cluster shows no sequence similarity to the Little Skate SINE fragment and it will be interesting to trace the evolution of this SINE within other members of the Rajidae. Repeatmasker [27] identifies 6523 bp (4.87%) of the Little Skate BAC clone as being repetitive, of which only 2432 bp (2.33%) is located in the gene-containing region between *HoxA1 *and *HoxA13*. The same region of the Horn Shark Hoxa cluster contains only 1205 bp (1.14%) of repetitive DNA, despite the larger genome size of Horn Shark. Aside from the SINE fragment in the Little Skate cluster, the rest of the repeats are simple-sequence elements such as mononucleotide tracts (see below) or microsatellites with a repeating unit of 2-6 bp. It seems likely therefore that the constraining force on repetitive DNA in vertebrate Hox clusters is not the preservation of precise intergenic distances (since microsatellites are known to expand and contract by strand slippage during DNA replication) but rather the exclusion of alternative transcriptional start sites in LINE or SINE promoters which may disrupt the tightly controlled expression of Hox genes during development.

Both the Skate and Horn Shark Hoxa clusters contain a number of guanine-rich sequences (Figure 5b). Regions such as this have been shown to be involved in transcriptional regulation of genes through the formation of stable four-stranded structures (the G-quadruplex or G4 DNA [28]) and it is possible that these sequences may be involved in transcriptional control of the Hoxa genes. The Quadparser program [29] identifies 13 putative G4-forming regions in the Little Skate BAC sequence, nine of which are located within the coding region between *Hoxa13 *and *Hoxa1*. The orthologus region in Horn Shark contains 14 such sequences and although some relative positions appear to be conserved (such as between *Hoxa9 *and *Hoxa10 *and a doublet between *Hoxa4 *and *Hoxa5*) these do not appear to be homologous based on conservation of both the G4 quadruplex and surrounding sequences. Because of this, it appears unlikely that G4 DNA plays an ancestral role in Hoxa gene regulation, although it is still possible that there are some cell- or species-specific requirements for the G4 structures in non-embryonic Hox expression.

## Conclusion

The Little Skate Hoxa cluster is strikingly similar to that of the Horn Shark in terms of both sequence conservation and intergenic distances despite the two lineages having been separated by at least 180 million years. The available data from the ongoing Elephant Shark (*Callorhinchus milii*) genome project suggests that the Hoxa cluster of this species is also highly conserved with respect to the Skate and Horn Shark despite having been separated from them for around 375 million years. Comparisons of the human Hoxa cluster to those of marsupials and amphibians reveal a much lower level of sequence conservation over similar periods of time, indicating either a faster rate of molecular evolution in the tetrapod and mammalian lineages or a slower rate of evolution in the cartilaginous fish. This finding echoes the suggestion of Wang *et al*. [21] that UCEs and protein-coding genes are evolving more slowly in Elephant Shark compared to other vertebrates. If the level of conservation seen in the chondrichthyan Hoxa clusters in reflected across the entire genome then this will complicate attempts to identify functional conserved non-coding elements within the cartilaginous fish using phylogenetic footprinting. This is an important consideration given the two cartilaginous fish genomes currently in the pipeline and the likely increase in this number due to recent advances in genome sequencing technologies.

## Methods

The Little Skate BAC clone 0081H20 was isolated in a low stringency pooled homeobox screen of a set of 4.1× coverage BAC library (BAC Library RE__Ba (Little Skate)) filters from the Clemson University Genomics Institute (CUGI). The BAC was shotgun sequenced to 9.3× coverage by contract to the Genome Center at Washington University, resulting in 14 major contigs in 4 scaffolds. Remaining gaps were filled using a combination of direct sequencing on BAC DNA or by cloning and sequencing of PCR products from primers designed to cross gaps in the scaffolds. Genes were annotated by comparison to published Horn Shark genes (from BAC sequences described by [3] and [4] deposited under accession numbers AF224262 and AF479755). Chondrichthyan non-coding elements were identified using the LAGAN alignment program [30] implemented in mVISTA [31]. Putative ncRNAs were identified using Infernal (version 1.0rc3) [32] and covariance models [33] of miRNA families from Rfam (version 9.1) [34]. Candidate precursors of microRNAs were selected with sequence length greater than 60 nt and minimum free energy of stem-loop structure less than -20 kcal/mol. RNA structures were drawn using the RNAfold (Vienna RNA Package 1.8.2) webserver .

## Authors' contributions

JFM carried out the experiments, JFM, YFZ and PWHH analysed the data and JFM and PWHH wrote the paper. All authors read and approved the final manuscript.
